# Low eGFR Is a Strong Predictor of Worse Outcome in Hospitalized COVID-19 Patients

**DOI:** 10.3390/jcm10225224

**Published:** 2021-11-09

**Authors:** Antonio Mirijello, Pamela Piscitelli, Angela de Matthaeis, Michele Inglese, Maria Maddalena D’Errico, Valentina Massa, Antonio Greco, Andrea Fontana, Massimiliano Copetti, Lucia Florio, Maurizio Angelo Leone, Michele Antonio Prencipe, Filippo Aucella, Salvatore De Cosmo

**Affiliations:** 1 Unit of Internal Medicine, Department of Medical Sciences, IRCCS Casa Sollievo della Sofferenza, 71013 San Giovanni Rotondo, Italy; pamela.piscitelli@gmail.com (P.P.); angeladematthaeis@gmail.com (A.d.M.); nglmhl@yahoo.it (M.I.); 2 Unit of Geriatrics, Department of Medical Sciences, IRCCS Casa Sollievo della Sofferenza, 71013 San Giovanni Rotondo, Italy; mm.derrico@gmail.com (M.M.D.); valinamas@hotmail.it (V.M.); a.greco@operapadrepio.it (A.G.); 3 Unit of Biostatistics, IRCCS Casa Sollievo della Sofferenza, 71013 San Giovanni Rotondo, Italy; a.fontana@operapadrepio.it (A.F.); m.copetti@operapadrepio.it (M.C.); 4 Unit of Neurology, Department of Medical Sciences, IRCCS Casa Sollievo della Sofferenza, 71013 San Giovanni Rotondo, Italy; l.florio@operapadrepio.it (L.F.); m.leone@operapadrepio.it (M.A.L.); 5 Unit of Nephrology, Department of Medical Sciences, IRCCS Casa Sollievo della Sofferenza, 71013 San Giovanni Rotondo, Italy; ma.prencipe@operapadrepio.it (M.A.P.); f.aucella@operapadrepio.it (F.A.)

**Keywords:** chronic kidney disease, glomerular filtration rate, respiratory failure, cardiovascular comorbidities

## Abstract

Background: The clinical course of COVID-19 is more severe in elderly patients with cardio-metabolic co-morbidities. Chronic kidney disease is considered an independent cardiovascular risk factor. We aimed to evaluate the impact of reduced eGFR on the composite outcome of admission to ICU and death in a sample of consecutive COVID-19 hospitalized patients. Methods: We retrospectively evaluated clinical records of a consecutive sample of hospitalized COVID-19 patients. A total of 231 patients were considered for statistical analysis. The whole sample was divided in two groups on the basis of eGFR value, e.g., ≥ or <60 mL/min/1.73 m^2^. Patients with low eGFR were further divided among those with a history of chronic kidney disease (CKD) and those without (AKI, acute kidney injury). The primary outcome was a composite of admission to ICU or death, whichever occurred first. The single components were secondary outcomes. Results: Seventy-nine (34.2%) patients reached the composite outcome. A total of 64 patients (27.7%) died during hospitalization, and 41 (17.7%) were admitted to the ICU. A significantly higher number of events was present among patients with low eGFR (*p* < 0.0001). Age (*p* < 0.001), SpO2 (*p* < 0.001), previous anti-platelet treatment (*p* = 0.006), Charlson’s Comorbidities Index (*p* < 0.001), serum creatinine (*p* < 0.001), eGFR (*p* = 0.003), low eGFR (*p* < 0.001), blood glucose levels (*p* < 0.001), and LDH (*p* = 0.003) were significantly associated with the main outcome in univariate analysis. Low eGFR (HR 1.64, 95% CI 1.02–2.63, *p* = 0.040) and age (HR per 5 years 1.22, 95% CI 1.10–1.36, *p* < 0.001) were significantly and independently associated with the main outcome in the multivariate model. Patients with AKI showed an increased hazard ratio to reach the combined outcome (*p* = 0.059), while those patients with both CKD had a significantly higher probability of developing the combined outcome (*p* < 0.001). Conclusions: Patients with reduced eGFR at admission should be considered at high risk for clinical deterioration and death, requiring the best supportive treatment in order to prevent the worst outcome.

## 1. Introduction

Clinical features of the coronavirus disease 2019 (COVID-19) vary from asymptomatic to fulminant cases with a subset of patients developing the severe disease characterized by respiratory failure, multi-organ dysfunction, the need for ventilatory and cardiac support, and death [1,2]. At present, older age and cardio-metabolic co-morbidities (e.g., diabetes, hypertension, obesity) are thought to be the main risk factors for a worse outcome [3,4,5]. Consequently, there is a need to identify predictors of the worst outcome in order to optimize treatment strategies. 

Renal function loss represents a para-physiological phenomenon secondary to aging [6], with a reduction of the glomerular function rate (GFR) of about 1 mL/min per year [7]. In addition, metabolic co-morbidities, such as diabetes mellitus and hypertension, negatively impact renal function, inducing a faster GFR loss [8]. Given the strict association with cardiovascular (CV) disease, chronic kidney disease (CKD) is nowadays considered a CV risk factor itself [9].

Reduced GFR, together with albuminuria, represents a key feature of CKD [10]. It has been demonstrated that a reduction of the estimated GFR (eGFR) is associated with increased mortality in the general population, independently from CKD [11]. In addition, low GFR characterizes acute kidney injury (AKI), defined as an abrupt decrease in kidney function, due to both structural damage and functional impairment. AKI is frequent among patients with sepsis [12]. 

A significant prevalence of renal dysfunction (e.g., elevated serum creatinine, elevated blood urea nitrogen, reduced eGFR) has been described during the course of the COVID-19 disease [13,14]. In this context, several factors could negatively impact the renal function of COVID-19 patients, such as fever, systemic inflammation, hypovolemia, low cardiac output, shock, and ventilation protocols [15,16]. Moreover, an association between acute cardiac injury and acute kidney injury has been described among severe COVID-19 patients [16].

At present, most of the evidence suggests a negative impact of reduced GFR on the course and outcome of COVID-19, with an increased risk of admission to the Intensive Care Unit (ICU) [16] and death [4,5,14,15,17,18,19] among patients with reduced renal function. However, not all studies confirmed this association [20].

The aim of the present study was to evaluate the impact of reduced eGFR (i.e., eGFR < 60 mL/min/1.73 m^2^) on the composite outcome of admission to ICU and death in a sample of consecutive COVID-19 hospitalized patients.

## 2. Materials and Methods

### 2.1. Patients

During the first pandemic wave (from March to May 2020), the COVID-19 Units of our tertiary-care hospital managed a total of 254 patients admitted for suspected SARS-CoV-2 infection. All admitted patients had epidemiological, clinical, laboratory, and radiologic findings suspected for COVID-19 [21]. Real-time reverse-transcriptase-polymerase-chain-reaction (RT-PCR) from a naso-pharyngeal swab was performed in all patients, and repeated, in the case of a negative result, as appropriate, according to guidelines [22]. As already described, 169 patients had a swab-confirmed diagnosis, 26 diagnoses were antibody-confirmed, and 37 patients, despite negative swabs, showed typical clinical and radiological features of COVID-19. A total of 22 patients were considered non-COVID and excluded from the sample due to an alternative diagnosis [21]. The Ethics Committee of our Institution approved the study (COVID-19-CSS, num. 46/2020, 8 April 2020). Patients gave their informed consent to participate. For patients unable to give their consent or those deceased, only the collection of data from clinical records was allowed. The study was conducted according to the Declaration of Helsinki.

### 2.2. Methods

Demographic characteristics (age, gender, education, marital status, employment, BMI, smoking status), medical history (onset of symptoms, date of admission, chronic diseases, and medications), and baseline clinical information (blood pressure, heart rate, peripheral oxygen saturation (SpO2), respiratory rate, body temperature, and Glasgow Coma scale (GCS)) were recorded at admission. The Charlson comorbidity index (CCI) [23] was also calculated. All patients underwent a standardized diagnostic workup at admission (creatinine, complete blood count, blood gas analysis, C-reactive protein, procalcitonin, lactates, D-dimer, troponin, ferritin, creatin-kinase, fibrinogen, interleukin-6, sputum, blood and urine culture, exclusion of other causes of interstitial pneumonia, and electrocardiogram). Anonymized data were recorded in electronic clinical record forms. 

Inclusion criteria for the present study were age ≥ 18-year-old and SARS-CoV-2 infection. Exclusion criteria were denied consent or lack of information on renal function. 

**Figure 1:**
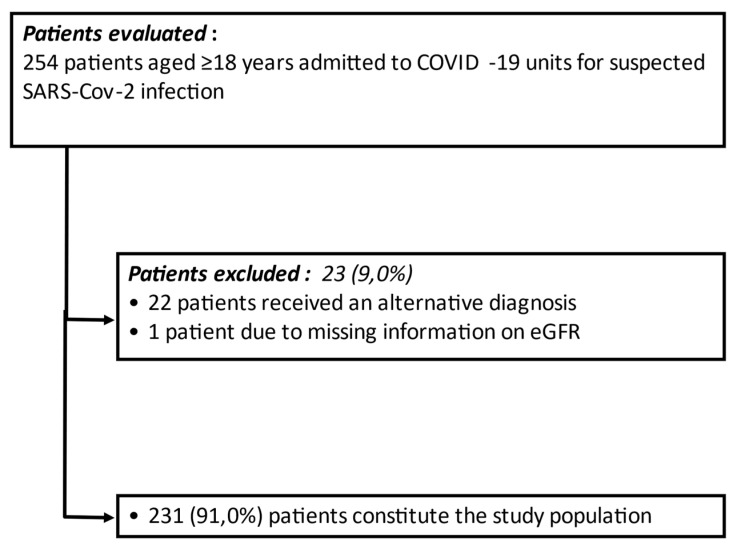
The number of patients evaluated for study inclusion, the number of patients excluded from the study with relative reason, and the number of patients included in the study.

Among the 254 patients admitted for suspected SARS-CoV-2 infection, a total of 23 (9.0%) were excluded for the reasons shown in Figure 1. Thus, a total of 231 (91.0%) patients met inclusion/exclusion criteria and were considered for statistical analysis.

GFR was estimated using a standardized serum creatinine assay and the Chronic Kidney Disease Epidemiology Collaboration formula [24]. For the purpose of the study, the sample was divided in two groups on the basis of eGFR value, e.g., ≥ or <60 mL/min/1.73 m^2^. Moreover, patients with reduced eGFR were further divided based on the presence/absence of pre-existing CKD. AKI was defined as eGFR < 60 mL/min/1.73 m^2^ and no previous CKD history. 

### 2.3. Outcomes

The primary outcome of the present study was a composite of admission to ICU or death, whichever occurred first. The single components were secondary outcomes.

### 2.4. Statistical Analysis

Baseline patients’ characteristics were reported overall and by eGFR status (≥ or <60 mL/min/1.73 m^2^) as the mean and standard deviation (SD) or median (range) for continuous variables, and as frequencies and percentages for categorial variables. Group comparisons were carried out using the Mann–Withney U-test and the Pearson Chi-squared test for continuous and categorical variables, respectively. 

The duration to the main outcome was calculated from hospitalization to the occurrence of ICU admission or death, whichever occurred first. For patients not showing an event, the follow-up period ended at hospital discharge. Outcome free curves were estimated by using the Kaplan–Meier method and compared by the log-rank test. Univariable and multivariable proportional hazards Cox regression models were estimated, and risks were reported as hazard ratios (HR) along with their 95% confidence intervals (95% CI). The proportional hazard assumption was tested using Schoenfeld residuals. Covariates’ stepwise selection was performed in the multivariable analysis. A two-sided *p* value < 0.05 was considered for statistical significance. All statistical analyses were performed using the computing environment R [25].

## 3. Results

Table 1 shows the main clinical features of the 231 patients included in this report, as the whole population and divided according to eGFR categories (below/equal or above 60 mL/min/1.73 m^2^). The mean age of the whole sample was 68.6 ± 15.0 years. A significant proportion of patients showed comorbidities, including arterial hypertension (47.2%), type 2 diabetes (T2DM) (19.0%), dementia (17.7%), atrial fibrillation (14.3%), dyslipidemia (10.4%), and COPD (10.8%). 

At admission, the mean eGFR value was 88.6 ± 45.3 mL/min/1.73 m^2^ with a total of 63 patients (27.3%) showing eGFR below 60 mL/min/1.73 m^2^. Among patients presenting with low eGFR at admission, 39 out of 63 (61.9%) had a history of CKD, while 24 (38.1%) had no history of CKD, thus they were considered as having an AKI.

Seventy-nine (34.2%) patients reached the composite outcome. A total of 64 patients (27.7%) died during hospitalization, while 41 (17.7%) were admitted to ICU due to worsening respiratory function. 

Patients with low eGFR were older, showed lower GCS, and higher CCI scores than those with eGFR ≥ 60 mL/min/1.73 m^2^ (Table 1). Moreover, in the former group, there was a higher percentage of patients with T2DM, hypertension, and dementia, as well as more patients on anti-hypertensive and anti-platelet treatments. The proportion of patients treated with ACEi/ARB was not different between subgroups. In addition, patients with eGFR < 60 mL/min/1.73 m^2^ showed significantly higher levels of C reactive protein (CRP), procalcitonin (PCT), D-dimer, and troponin. Finally, a significantly higher number of events was present among patients with low eGFR (Table 1).

In a univariate analysis model, age (*p* < 0.001), SpO2 (*p* < 0.001), previous anti-platelet treatment (*p* = 0.006), CCI (*p* < 0.001), serum creatinine (*p* < 0.001), eGFR (*p* = 0.003), low eGFR (*p* < 0.001), blood glucose levels (*p* < 0.001), and LDH (*p* = 0.003) were significantly associated with the main outcome (Table 2).

Results of univariate analysis for the single components of the main outcome (death or admission to ICU) are reported in Supplementary Tables S1 and S2, respectively. Among variables included in the model, those significantly associated with death were age (*p* < 0.001), CCI (*p* < 0.001), T2DM (*p* = 0.007), low eGFR (*p* = 0.003), blood glucose levels (*p* < 0.001), LDH (*p* < 0.001), and SpO2 (*p* < 0.001) (Supplementary Table S1). The variables significantly associated with admission to ICU were age (*p* = 0.045), CCI (*p* = 0.003), previous antiplatelet treatment (*p* = 0.002), creatinine (*p* = 0.025), eGFR (*p* = 0.007), low eGFR (*p* = 0.015), cholesterol (*p* = 0.007), and SpO2 (*p* < 0.001) (Supplementary Table S2).

In a multivariate model including those variables significantly associated with the main outcome at univariate evaluation, low eGFR (HR 1.64, 95% CI 1.02–2.63, *p* = 0.04) and age (HR 1.22, 95% CI 1.10–1.35, *p* < 0.001) were significantly and independently associated with the main outcome (Table 3).

**Figure 2:**
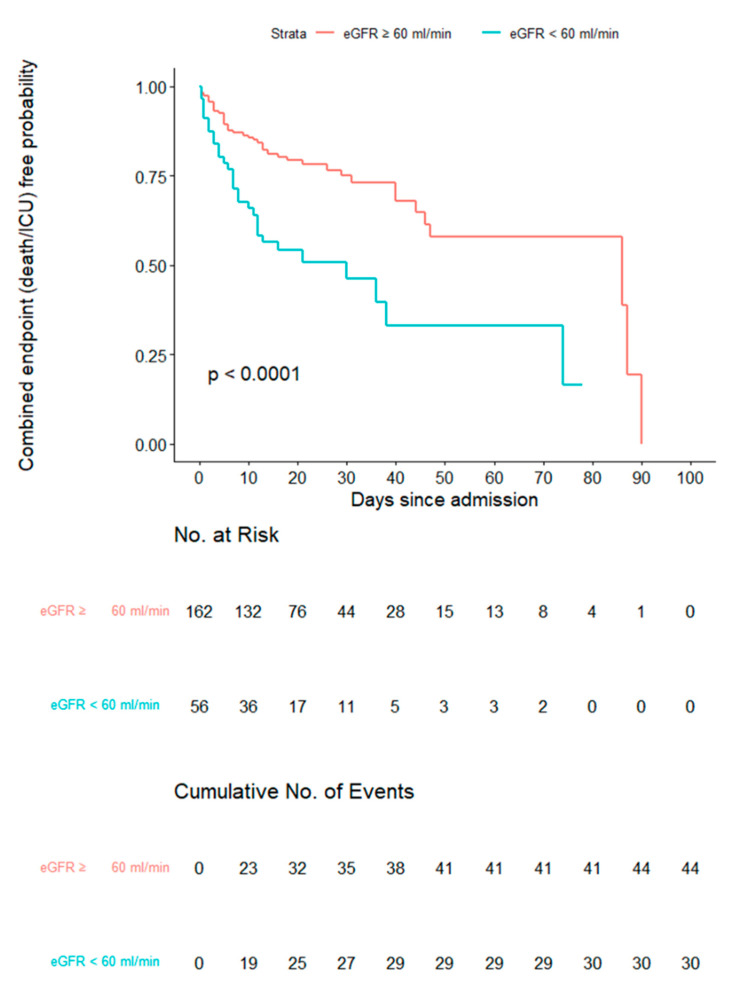
Kaplan–Meier survival curves for the main outcome (admission to ICU or death) in the pooled sample divided into two groups according to eGFR values (global log-rank test *p* < 0.0001).

The Kaplan–Meier survival curves for the main outcome in the pooled sample divided into two groups according to baseline eGFR values (e.g., ≥ or <60 mL/min/1.73 m^2^) are reported in Figure 2. A significantly higher rate of occurrence of the main outcome among patients with low eGFR was found (global log-rank test *p* < 0.001) (Figure 1).

Starting from the observation that patients with baseline eGFR < 60 mL/min/1.73 m^2^ showed a significantly higher relative risk to reach the combined endpoint with respect to patients with a baseline eGFR ≥ 60 mL/min/1.73 m^2^ (HR = 2.40; 95% CI = 1.53–3.76, *p* < 0.001) (Table 2), we further sub-grouped patients according to both baseline eGFR and previous history of CKD. We observed that, with respect to patients with baseline eGFR ≥ 60 mL/min/1.73 m^2^, those with AKI (low baseline eGFR and no history of CKD) showed a trend of an increased hazard ratio to reach the combined outcome (*p* = 0.059), while those patients with both low eGFR and CKD had a significantly higher probability to develop the combined outcome (*p* < 0.001) (Table 4). In addition, patients with both low eGFR and CKD had a higher risk of reaching the combined endpoint (HR = 1.44; 95% CI = 0.69–2.99, *p* = 0.3245) with respect to those with AKI (reference population), but this did not reach the statistically significant threshold. 

**Figure 3:**
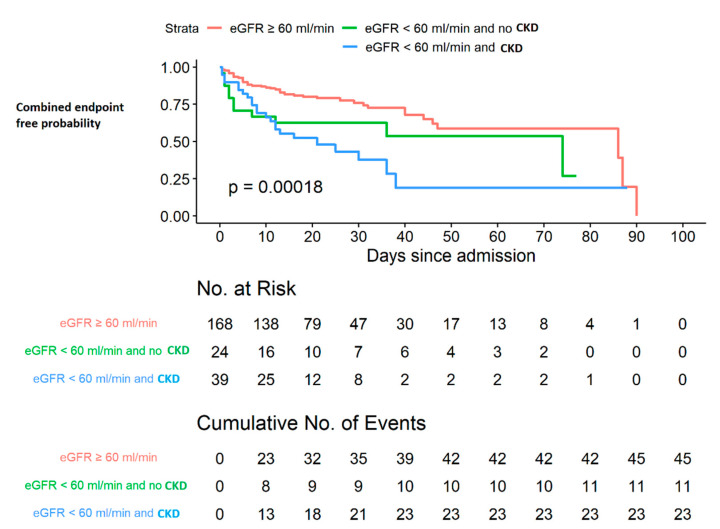
Kaplan–Meier survival curves for the main outcome (admission to ICU or death) in the pooled sample divided into three groups according to eGFR and CKD (global log-rank test *p* < 0.001).

The Kaplan–Meier survival curves for the main outcome in the pooled sample divided into three groups according to baseline eGFR values (e.g., ≥ or <60 mL/min/1.73 m^2^) and history of CKD are reported in Figure 3. A significant difference of occurrence of the main outcome among the three groups was found (global log-rank test *p* < 0.001) (Figure 3).

## 4. Discussion

The present study shows that among 231 patients hospitalized because of COVID-19 pneumonia, 34.2% developed a poor outcome (e.g., admission to ICU or death). It is clinically relevant to determine which COVID-19 patients will have a poor prognosis. In our study, low admission eGFR and older age were significant and independent predictors of the outcome. The findings of our study come from an Italian tertiary care Hospital that was included in the COVID-19 health network during the first pandemic wave, thus representing a real-life setting. 

We found a high prevalence of kidney dysfunction among COVID-19 patients at admission, with approximately a quarter (27.2%) of patients showing an eGFR < 60 mL/min/1.73 m^2^. Among these, the reason for low eGFR was CKD in 62% of cases and AKI in 38%. 

Our findings are in line with recent data from Uribarri et al., who reported 30% of patients with low eGFR (<60 mL/min/1.73 m^2^) on admission in a sample including 758 patients with SARS-CoV-2 infection [15]. However, our sample included a higher proportion of CKD patients [15]. In the cohort of Gok et al., including 609 consecutive adult patients hospitalized with a diagnosis of COVID-19 in a tertiary-level hospital, the percentage of patients with low eGFR was close to 21%, rather similar to our finding [18]. A quarter of the evaluated patients showed AKI [18]. 

Previous studies have shown that in patients with COVID-19 infection, the presence of chronic comorbidities, such as diabetes, arterial hypertension, or cardiovascular disease negatively influence the outcome [3,4,5]. The presence of CKD has been also reported by some authors to have a deleterious effect in patients with SARS-CoV-2 infection [4,26].

Our data confirm and expand this information in a different setting, showing an independently increased relative risk of 64% of poor outcomes among patients with eGFR below 60 mL/min/1.73 m^2^. This result suggests that patients with CKD should be aware of the risk they run by contracting the SARS-CoV-2 infection and, consequently, be particularly cautious to avoid the infection. Moreover, the presence of CKD should be fully considered in the risk stratification of COVID-19 patients.

The increased risk of mortality associated with CKD in patients with SARS-CoV-2 infection has been highlighted by Williamson E et al. [4]. These authors focused on the role of CKD, showing a graded association between the level of kidney dysfunction and the risk of COVID-19 mortality, which was highest in patients with the severest grade of CKD [4]. In line with these findings, Uribarri et al. showed an increased risk for in-hospital complications and mortality among patients with renal failure [15]. Similarly, according to Gok et al., both AKI and CKD are independently associated with mortality [18]. On the contrary, Bravi et al., did not find eGFR to be an independent predictor of poor outcomes. However, the studied population was younger and showed a lower prevalence of kidney dysfunction with respect to our sample [20].

Our study does not explore, by its nature, the reasons for the poor outcome in CKD patients. However, the result is independent from traditional risk factors such as diabetes, obesity, or hypertension, while non-traditional risk factors such as reduced immune response, increased susceptibility to infections, or clotting abnormalities cannot be excluded as playing a causative role.

In our analysis, we found significant clinical differences between the two analyzed groups (i.e., below or equal or above 60 mL/min/1.73 m^2^). In particular, patients with low eGFR were older and with more comorbidities (Table 1), such as T2DM, hypertension, and dementia. In addition, they showed a more severe degree of inflammation (e.g., CRP, PCT), myocardial damage (e.g., troponin), and fibrinolysis (e.g., D-dimer) with respect to those patients without low eGFR. These observations are in line with recent literature data, confirming the more severe course of COVID-19 disease among patients with CKD [4,19,26]. As per troponin and D-dimer, it should also be underlined that renal dysfunction could, at least in part, contribute to their raised levels, due to a reduced clearance. Contrarily to recent literature reports [27], we did not find differences among genders.

When we explored the variables associated with the single components of the main outcome, we interestingly found that the presence of diabetes and high CCI significantly increased the risk of death but not of ICU admission. However, the latter negative result could be biased by the relatively small sample size. In addition, among variables associated with the worst outcome, age and CCI reflect the burden of comorbidities and have been shown to predict mortality in COVID-19 patients [28], while being on antiplatelet treatment could be considered as a surrogate marker of increased CV risk. 

This study has some limitations. First, we do not have information on kidney function before virus infection and consequently we cannot exclude that kidney impairment was induced by the SARS-CoV-2 infection. Second, the number of patients enrolled, as well as the follow-up duration, was limited to the stay in hospital; thus, we do not have information on long-term infection outcomes. In addition, no formal calculation of the sample size was performed in the present study. As a consequence, the reduced number of patients showing an eGFR above 60 mL/min/1.73 m^2^ and the retrospective design could represent a further limitation. 

## 5. Conclusions

Respiratory failure represents the main reason for hospitalization in patients affected by COVID-19 [2]. Fast clinical worsening and the need for ventilatory support are the most feared complications by both patients and physicians, often configuring a dramatic succession of events leading to admission to ICU and death in a significant proportion of patients. As a consequence, the identification of predictors of the worst outcome is pivotal for the early stratification of risky patients and for optimal treatment strategies. Patients with reduced eGFR at admission should be considered at high risk for clinical deterioration and death, requiring the best supportive treatment in order to prevent the worst outcome.

## Figures and Tables

**Table 1:** Main clinical features of the 231 patients included in the study, as a whole population and divided according to eGFR categories (below/equal or above 60 mL/min/1.73 m^2^).

Main Patients’ Clinical Features	Whole Population (*n* = 231)	eGFR ≥ 60 mL/min/1.73 m^2^ (*n* = 168)	eGFR < 60 mL/min/1.73 m^2^ (*n* = 63)	*p* Value
Age (years old)	68.6 ± 15.0	65.3 ± 15.1	77.3 ± 10.4	<0.001
Gender (M/F)	125/106 (54.1%/45.9%)	97/71 (57.7%/42.3%)	28/35 (44.4%/55.6%)	0.071
BMI kg/m^2^	26.9 ± 4.6	26.7 ± 4.5	27.5 ± 5.0	0.328
GCS (points)	14.4 ± 2.1	14.6 ± 1.6	13.7 ± 3.1	0.003
CCI (points)	3.9 ± 2.5	3.2 ± 2.3	5.7 ± 2.0	<0.001
Diabetes, *n* (%)	44 (19.0%)	22 (13.1%)	22 (34.9%)	<0.001
Hypertension, *n* (%)	109 (47.2%)	67 (39.9%)	42 (66.7%)	<0.001
Dyslipidemia, *n* (%)	24 (10.4%)	16 (9.5%)	8 (12.7%)	0.938
Atrial Fibrillation, *n* (%)	33 (14.3%)	20 (11.9%)	13 (20.6%)	0.091
COPD, *n* (%)	25 (10.8%)	17 (10.1%)	8 (12.7%)	0.574
Dementia, *n* (%)	41 (17.7%)	20 (11.9%)	21 (33.3%)	<0.001
Antiplatelet treatment, *n* (%)	50 (21.6%)	27 (16.1%)	23 (36.5%)	<0.001
Anti-hypertensive treatment, *n* (%)	105 (45.4%)	62 (36.9%)	42 (66.7%)	<0.001
ACEi/ARB, *n* (%)	73 (31.6%)	48 (28.6%)	25 (39.7%)	0.106
Serum creatinine (mg/dL)	1.2 ± 1.2	0.7 ± 0.2	2.3 ± 1.9	<0.001
eGFR (mL/min/1.73 m^2^)	88.6 ± 45.3	108.4 ± 36.0	35.9 ± 15.5	<0.001
eGFR < 60 mL/min/1.73 m^2^	63 (27.2%)	-	-	-
CKD, *n* (%)	39 (16.9%)	-	39 (61.9%)	-
AKI, *n* (%)	24 (10.4%)	-	24 (38.1%)	-
Glycemia (mg/dL)	121.8 ± 56.2	110.8 ± 46.2	150.0 ± 71.0	<0.001
Triglycerides (mg/dL)	124.5 ± 52.8	123.1 ± 54.4	128.6 ± 48.4	0.354
Cholesterol (mg/dL)	138.7 ± 40.4	143.1 ± 40.3	126.3 ± 38.3	0.011
LDH (UI/L)	284.4 ± 160.9	275.5 ± 142.8	307.2 ± 199.9	0.997
SpO2 (%)	92.6 ± 5.7	92.9 ± 5.3	91.6 ± 7.0	0.598
Troponin (ng/mL)	238.7 ± 1580.1	63.2 ± 209.0	698.5 ± 2975.6	<0.001
D-dimer (ng/mL)	3325.0 ± 8053.6	2585.2 ± 6115.9	5409.9 ± 11821.7	0.013
CRP (mg/dL)	7.4 ± 7.2	6.7 ± 6.7	9.3 ± 8.2	0.035
PCT (ng/mL)	1.2 ± 4.0	0.9 ± 4.0	2.2 ± 4.0	<0.001
IL-6 (pg/mL)	73.0 ± 196.0	68.2 ± 196.2	87.9 ± 201.0	0.226
Occurrence of primary outcome, *n* (%)	79 (34.2%)	45 (26.8%)	34 (54.0%)	<0.001

Abbreviations: BMI: Body Mass Index; GCS: Glasgow Coma Scale; CCI: Charlson comorbidity index; COPD: Chronic obstructive pulmonary disease; ACEi: Angiotensin converting enzyme inhibitors; ARB: angiotensin receptor blockers; GFR: Glomerular filtration rate; eGFR: Estimated GFR; CKD: Chronic kidney disease; AKI: Acute kidney injury; LDH: lactate dehydrogenase; SpO2: saturation of peripheral oxygen; CRP: C reactive protein; PCT: Procalcitonin; IL-6: interleukin 6.

**Table 2:** Results of Cox univariate regression analysis for characteristics associated with the main outcome (death + admission to ICU).

Time to Combined Endpoint		
Variable	HR (95% CI)	*p* Value
Age (per 5 years)	1.26 (1.15–1.39)	<0.001
Gender (M/F)	1.04 (0.66–1.62)	0.871
BMI kg/m^2^	1.02 (0.97–1.07)	0.457
CCI (points)	1.26 (1.15–1.38)	<0.001
Diabetes, *n* (%)	1.58 (0.95–2.64)	0.079
Antiplatelet treatment, *n* (%)	1.96 (1.21–3.19)	0.006
Anti-dyslipidemia treatment, *n* (%)	0.94 (0.48–1.84)	0.854
Anti-hypertensive treatment, *n* (%)	1.20 (0.77–1.87)	0.427
Serum creatinine (mg/dL)	1.28 (1.15–1.44)	<0.001
eGFR (per 10 mL/min/1.73 m^2^)	0.93 (0.88–0.97)	0.003
eGFR < 60 mL/min/1.73 m^2^	2.40 (1.53–3.76)	<0.001
Blood glucose (mg/dL)	1 (1–1.01)	<0.001
Triglycerides (mg/dL)	1 (1–1.01)	0.853
Cholesterol (mg/dL)	0.99 (0.99–1)	0.115
LDH (UI/L)	1 (1–1)	0.003
SpO2 (%)	0.93 (0.89–0.96)	<0.001

Abbreviations: BMI: Body Mass Index; GCS: Glasgow Coma Scale; CCI: Charlson comorbidity index; GFR: Glomerular filtration rate; eGFR: Estimated GFR; LDH: lactate dehydrogenase; SpO2: saturation of peripheral oxygen.

**Table 3:** Results of Cox multivariate regression analysis for variables significantly and independently associated with main outcome. Abbreviations: eGFR: Estimated GFR.

Variable	HR (95% CI)	*p* Value
Age (per 5 years)	1.22 (1.10–1.35)	<0.001
eGFR < 60 mL/min/1.73 m^2^	1.64 (1.02–2.63)	0.040

**Table 4:** Risk for developing the composite outcome according to AKI and CKD, considering baseline eGFR ≥ 60 mL/min/1.73 m^2^ as reference (Cox regression analysis).

Group	HR	*p* Value
Baseline eGFR ≥ 60 mL/min/1.73 m^2^	1	n.a.
AKI	1.89 (0.97–3.68)	0.059
CKD	2.59 (1.64–4.54)	<0.001

Abbreviations: AKI: Acute kidney injury; CKD: Chronic kidney disease.

## Data Availability

Data supporting the reported results may be provided on reasonable request.

## Supplementary Materials

The following are available online at https://www.mdpi.com/article/10.3390/jcm10225224/s1, Table S1: Results of Cox univariate regression analysis for variables associated with death, Table S2: Results of Cox univariate regression analysis for characteristics associated with admission to ICU.
