# Supplementary material for: Low eGFR Is a Strong Predictor of Worse Outcome in Hospitalized COVID-19 Patients
Source: J Clin Med. 2021 Nov 9;10(22):5224. doi: 10.3390/jcm10225224 (PMC8619033; doi:10.3390/jcm10225224)
Supplement: Supplementary file 1 [file jcm-10-05224-s001.zip › jcm-1453349-supplementary.pdf]

Supplementary Materials

Table S1. Results of Cox univariate regression analysis for variables associated with death (abbreviations: BMI: Body Mass Index; CCI: Charlson comorbidity index; GFR: Glomerular filtration rate; eGFR: Estimated GFR).

Time to Death		
**Variable**	**HR (95% CI)**	***p* Value**
Age (per 5 years)	1.33 (1.18–1.49)	**<0.001**
Gender (M/F)	1.10 (0.67–1.81)	0.699
BMI kg/m^2	1.03 (0.97–1.08)	0.357
CCI (points)	1.33 (1.20–1.48)	**<0.001**
Diabetes, *n* (%)	2.12 (1.23–3.64)	**0.007**
Antiplatelet treatment, *n* (%)	1.76 (1.02–3.03)	**0.041**
Anti-hypertensive treatment, *n* (%)	1.26 (0.77–2.06)	0.366
Serum creatinine (mg/dl)	1.12 (0.98–1.28	0.093
eGFR (per 10 mL/min/1.73 m^2)	0.95 (0.90–1.00)	0.071
eGFR ≤ 60 mL/min/1.73 m^2	2.12 (1.29–3.50)	**0.003**
Glycemia (mg/dl)	1.01 (1–1.01)	**<0.001**
Triglycerides (mg/dl)	1 (1–1.01)	0.350
Cholesterol (mg/dl)	1 (0.99–1.01)	0.889
LDH (UI/l)	1 (1–1)	**<0.001**
SpO2 (%)	0.91 (0.88–0.95)	**<0.001**

Table S2. Results of Cox univariate regression analysis for characteristics associated with admission to ICU (abbreviations: BMI: Body Mass Index; CCI: Charlson comorbidity index; GFR: Glomerular filtration rate; eGFR: Estimated GFR).

Time to ICU		
**Variable**	**HR (95%CI)**	***p* Value**
Age (per 5 years)	1.02 (1–1.05)	**0.045**
Gender (M/F)	1.7 (0.89–3.25)	0.105
BMI kg/m^2	1.04 (0.97–1.11)	0.242
CCI (points)	1.15 (1.02–1.29)	**0.003**
Diabetes, *n* (%)	1.21 (0.58–2.53)	0.619
Antiplatelet treatment, *n* (%)	2.60 (1.39–4.87)	**0.002**
Anti-hypertensive treatment, *n* (%)	1.50 (0.81–2.77)	0.988
Serum creatinine (mg/dl)	1.38 (1.20–1.58)	**0.025**
eGFR (per 10 mL/min/1.73 m^2)	0.90 (0.84–0.97)	**0.007**
eGFR ≤ 60 mL/min/1.73 m^2	2.15 (1.16–4.01)	**0.015**
Glycemia (mg/dl)	1 (1–1.01)	0.058
Triglycerides (mg/dl)	1 (0.99–1)	0.467
Cholesterol (mg/dl)	0.99 (0.97–1)	**0.007**
LDH (UI/l)	1 (1–1)	0.057
SpO2 (%)	0.92 (0.88–0.96)	**<0.001**
